# Beyond “Monologicality”? Exploring Conspiracist Worldviews

**DOI:** 10.3389/fpsyg.2017.00861

**Published:** 2017-06-20

**Authors:** Bradley Franks, Adrian Bangerter, Martin W. Bauer, Matthew Hall, Mark C. Noort

**Affiliations:** ^1^Department of Psychological and Behavioural Science, London School of Economics and Political ScienceLondon, United Kingdom; ^2^Institut de Psychologie du Travail et des Organisations, Université de NeuchâtelNeuchâtel, Switzerland

**Keywords:** conspiracy theories, monological belief system, worldviews, quasi-religion, interviews

## Abstract

Conspiracy theories (CTs) are widespread ways by which people make sense of unsettling or disturbing cultural events. Belief in CTs is often connected to problematic consequences, such as decreased engagement with conventional political action or even political extremism, so understanding the psychological and social qualities of CT belief is important. CTs have often been understood to be “monological,” displaying the tendency for belief in one conspiracy theory to be correlated with belief in (many) others. Explanations of monologicality invoke a nomothetical or “closed” mindset whereby mutually supporting beliefs based on mistrust of official explanations are used to interpret public events as conspiracies, independent of the facts about those events (which they may ignore or deny). But research on monologicality offers little discussion of the content of monological beliefs and reasoning from the standpoint of the CT believers. This is due in part to the “access problem”: CT believers are averse to being researched because they often distrust researchers and what they appear to represent. Using several strategies to address the access problem we were able to engage CT believers in semi-structured interviews, combining their results with analysis of media documents and field observations to reconstruct a conspiracy worldview – a set of symbolic resources drawn on by CT believers about important dimensions of ontology, epistemology, and human agency. The worldview is structured around six main dimensions: the nature of reality, the self, the outgroup, the ingroup, relevant social and political action, and possible future change. We also describe an ascending typology of five types of CT believers, which vary according to their positions on each of these dimensions. Our findings converge with prior explorations of CT beliefs but also revealed novel aspects: A sense of community among CT believers, a highly differentiated representation of the outgroup, a personal journey of conversion, variegated kinds of political action, and optimistic belief in future change. These findings are at odds with the typical image of monological CT believers as paranoid, cynical, anomic and irrational. For many, the CT worldview may rather constitute the ideological underpinning of a nascent pre-figurative social movement.

## Introduction

Explaining complex societal events is itself complex. The use of conspiracy theories (CTs) to make sense of destabilizing events (like the assassination of major public figures, the unpredicted destruction of major public buildings, sudden infectious disease outbreaks) is a widespread response to world complexity (e.g., van Prooijen, 2011). CTs involve symbolic coping which transmutes the diffuse anxiety arising from such events into specific threats caused by the purportedly malevolent action of powerful actors (e.g., Harrison and Thomas, 1997; Wagner-Egger and Bangerter, 2007; Byford, 2011). Since societal complexity and uncertainty appear to be increasing, conspiratorial thinking may increase as a response (e.g., Aupers, 2012).

A range of practical consequences of belief in CTs has been documented. For example, exposure to anti-vaccine CTs decreases people’s intentions to vaccinate (Jolley and Douglas, 2014a). Similar society-wide public health implications arose for polio vaccination in Nigeria (Falade and Bauer, 2017), where the vaccine was seen as the instrument of a Western birth-control plot. In the United States, belief that birth control and HIV/AIDS are forms of genocide against African Americans is associated with negative attitudes toward contraception (Bogart and Thorburn, 2006). In broader terms, CT belief and exposure is associated with feelings of powerlessness (Abalakina-Paap et al., 1999; Jolley and Douglas, 2014b), which, for specific anti-government and climate change CTs decreases conventional political engagement and pro-environmental intentions. Moreover, CT belief correlates with political extremism (van Prooijen et al., 2015), and generalized CT beliefs have been argued to be precursors of terrorism-endorsing beliefs (Bartlett and Miller, 2010).

Since belief in CTs has significant practical consequences, it is important to understand their associated psychological and social factors. Psychological qualities associated with CT belief include Machiavellianism (Douglas and Sutton, 2011), schizotypy (Darwin et al., 2011), anomie, political cynicism, distrust in authority, (Goertzel, 1994; Abalakina-Paap et al., 1999; Swami et al., 2011). Specific aspects of cognitive processing associated with CT belief include higher tendency to detect agency where there may be none (e.g., Brotherton and French, 2015), which is reduced by higher levels of education (Douglas et al., 2016), an effect that may be explained by the general negative correlation between belief in CTs and analytic processing style (Swami et al., 2014). CT belief is also associated with processing errors and biases – such as the conjunction fallacy (tendency to overestimate the probability of co-occurring events: Brotherton and French, 2015), and the proportionality bias (attributing larger-scale causes to more significant events: Leman and Cinnirella, 2007). However, belief in CTs has also been found to be somewhat responsive to circumstances: van Prooijen and Jostmann (2013) found that inducing uncertainty increased conspiracy belief, whilst exposure to specific CTs (e.g., concerning Princess Diana: Douglas and Sutton, 2008; and John F Kennedy: Butler et al., 1995) also increased it.

This paper offers two contributions to the study of CTs – one theoretical, based on empirical data; and another methodological. The theoretical contribution concerns the contention that CTs are ‘monological’ (e.g., Swami et al., 2011; Wood et al., 2012; Sutton and Douglas, 2014): belief in one CT predicts belief in more CTs, providing the foundation of generalized conspiratorial perspective. We suggest that assessing the nature and degree of monologicality requires understanding the detailed contents of a *conspiratorial worldview*. The methodological contribution flows from the theoretical contribution; it concerns how researchers can access such contents given that CT believers are a “hard to access” population (Wood and Douglas, 2015). Sustained theoretical development and empirical assessment of monologicality requires addressing the methodological access problem.

Monologicality suggests that CT thinking is a stable cognitive style, disposition or trait. This possibility was outlined by Goertzel’s (1994) suggestion that conspiratorial thinking offers a general set of assumptions about authority “cover ups” which are portable across specific topics or events. Social psychology findings also suggest a “monological” tendency, whereby belief in one conspiracy predicts belief in others. CTs may thus comprise a network of mutually supporting beliefs about the functioning of the social world (Swami et al., 2011; Wood et al., 2012; Sutton and Douglas, 2014). Belief in CTs, on this account, is driven less by the specific contents of each CT and more by a general conspiratorial mentality (Moscovici, 1987) or worldview (Koltko-Rivera, 2004), whose main tenet involves rejection of official explanations. This suggests that CT belief does not arise from inferences drawn from a set of observations, but rather from applying a conspiratorial worldview to those observations. Indeed, Goertzel’s (1994, p. 739) original suggestion was that monological CTs expressed a “closed” mind, unlike “dialogical” belief systems, which “engage in a dialog with their context.” He also suggested that CTs need not be monological – some may be dialogical, if they are open to facts and disconfirming evidence.

Similarly, Sutton and Douglas (2014) pointed out several related problems with monologicality. The idea that CT belief indicates closed mindedness is contradicted by its correlating with openness to experience (Swami et al., 2010). The idea that CT believers are politically cynical is contradicted by the finding that CT belief sometimes correlates with support for democratic principles (Swami et al., 2011). Moreover, people can hold mutually contradictory CT beliefs, suggesting that monologicality is less driven by the CT accounts *per se* than by a more general belief in the deceptive nature of official explanations. Monological explanation also lacks parsimony, since third variables (e.g., personality traits) may affect belief in various conspiracies, creating spurious correlations between them.

Pursuing these issues, we contend that previous discussions of monologicality have had little to say about the *contents* of a putative conspiratorial worldview. They typically sample members of student and general populations using questionnaires to investigate their degree of belief in CTs formulated as vignettes by the researchers: monologicality is defined as the degree of correlation between belief in multiple CTs. Such correlations are then correlated with other psychological variables, offering an important picture of the structural landscape of CTs and those variables, as noted above. However, it is a landscape that is only sparsely populated by people’s concerns and the contents of their beliefs. Most social psychology research on monologicality in CTs is thus deliberately content-free, offering little account of the symbolic resources – worldviews – people draw on in constructing CTs and their use in everyday sensemaking [exceptions include Byford’s (2011) critical account of CTs, and Lewis and Kahn’s (2005) description of the cosmogony of CT guru David Icke]. This inattention to symbolic content is surprising given that one key function of CTs is precisely to make symbolic sense of destabilizing events, which can allow individuals and groups to cope with them.

Such past findings hint that individuals may adhere to a conspiratorial worldview to varying degrees. This might explain some of the conflicting findings indicated by Sutton and Douglas (2014). However, it remains unclear how individuals use elements of a conspiratorial worldview in sensemaking. More open-ended methods (such as semi-structured interviews) would allow participants to frame the content and degree of commitment to CTs on their own terms and out of their concrete life situations. They thereby allow finer-grained assessments of the content of conspiratorial mentality and degree of monologicality. The extent of their monologicality would emerge from their own descriptions of their beliefs rather than from interpreting their endorsement of a series of pre-determined items. And further insight would be gained into the symbolic foundations of a conspiratorial worldview. For example, epistemically, does a person believe all of the CTs they believe in the same way – the same level of conviction, responding to the same kinds of doubts about societal events and threats, offering similar degrees of uncertainty management? And ontologically, do the CTs all draw on the same everyday commonsense ontology, or do they posit entities or properties that go beyond the everyday, perhaps positing a role for the supernatural?

However, any such research project is confronted by the “access problem”: Wood and Douglas (2015, p. 6) note, people “with a high degree of conspiracist ideation” are likely to be averse to social science research, which is often associated with universities that are “part of the problem”: distrust of authority applies to universities as much as to governments and corporations. The London School of Economics, for example, takes a prominent role in David Icke’s conspiracist worldview (Vice, 2012).

The possibility that not all CT believers are monological, and that those who are may ground their monologicality in contents that are not confined to distrust of authority, flows from the quasi-religious approach to CTs (Franks et al., 2013). This suggests that CTs may be analogous to religious representations, involving explanations which use representations of conspiratorial actors with supernatural or super-human degrees of agency that reflect minimally counter-intuitive departures from commonsense explanations. These representations are communicated and reconstructed as part of the social sensemaking process, as in social representations theory (Bauer and Gaskell, 1999).

## Our Study

Against this backdrop, we aimed to document contents of CTs and link them to their use in sensemaking and symbolic coping by different individuals. These contents constitute materials for reconstructing a conspiracy worldview, as well as the potentially different ways in which individuals might subscribe to or engage with it. According to Koltko-Rivera (2004, p. 3), a worldview is a set of “beliefs and assumptions that describe reality. A given worldview encompasses assumptions about a heterogeneous variety of topics, including human nature, the meaning and nature of life, and the composition of the universe itself, to name but a few issues.” Therefore, a *conspiracy worldview* should involve positionings on issues of ontology (the nature of reality), epistemology (the nature of knowledge, what can be known), and agency (human action and free will) (Koltko-Rivera, 2004). Moreover, a specifically *conspiracy* worldview might also offer resources for self-definition, enabling believers to make sense of their life situation by positioning themselves relative to society and reality, suggesting (following Moscovici, 1987) that it involves representations of *society*, featuring distinctions between groups, especially (pure, good) ingroups and (malevolent) outgroups. Byford (2011) has analyzed the “anatomy” of CTs as narratives and identified elements including conspiratorial groups, conspiratorial plans and motifs like “manipulation of the many by the few.” Additionally, a conspiratorial worldview might function as a “meta-narrative” (Lyotard, 1979) that grounds individual CT stories.

We pursued this goal in a research project featuring open-ended collection and triangulation of qualitative data (most prominently discursive productions) from multiple sources (cultural products, participant observation, and semi-structured interviews) supported by thematic analysis over the course of two and a half years (May 2013 to December 2015). Like many qualitative endeavors (Golden-Biddle and Locke, 2007), our findings emerged progressively out of this process. The main implication of our study is a tentative typology along a spectrum of conspiracy worldviews and the thematic dimensions that constitute them. This typology is informed by our empirical data as well as by theoretical insights from relevant domains of social psychology, sociology, and anthropology.

### Data Collection

To investigate the detailed contents of the CT worldview, we sought to engage CT believers in interviews, and had to address the access problem, unlike past research which, in using student or general population samples, or written and on-line media, has thereby sought to circumvent it.

Recruitment of participants took place in two stages. These stages were not prospectively planned: Stage 2 emerged from the challenges arising in Stage 1. Stage 1 corresponds to a more informal, explorative moment, whereas in Stage 2, we conducted more formal data collection (interviews according to a specific sampling strategy). In Stage 1, we aimed to approach CT believers to understand the contents of their beliefs, and document some of the cultural products that circulate in their milieu (in websites, podcasts and their transcripts, books and mass media). As might be expected (Wood and Douglas, 2015), accessing participants was not straightforward: Several direct attempts failed. Individuals contacted via website chat-rooms dedicated to CTs^1^ were unwilling to be interviewed by university researchers, and one of us (MN) was subsequently excluded from those chat-rooms. Similarly, MB attempted to make contact with a CT source via a personal contact acting as middleman; contact was refused because the London School of Economics was deemed “part of the conspiratorial world.” Again, MH’s invitation for an interview was rejected on the grounds that the team of which he was a part were co-authors of a paper the respondent had read and considered to misrepresent those with conspiracy beliefs (Franks et al., 2013). A final example arose at a protest gathering outside the Bilderberg Group meeting in Watford, United Kingdom (June 8–9, 2013). After speaking with a participant, MN asked them to take part in an interview on CTs; this resulted in his being threatened and physically assaulted. These altercations echo the “recursive fury” over scientific analysis of conspiracist ideation (Lewandowsky et al., 2013).

We nevertheless managed to recruit one respondent for an interview via the Icke website as well as two other respondents via a personal relation of MB. All three respondents attended a presentation by David Icke at Wembley Stadium, United Kingdom. The couple was subsequently interviewed at their home abroad. We also engaged in participant observation at two conspiracy theory-related events – the protest meeting outside the Bilderberg Group meeting and a protest outside the Royal Courts of Justice in London regarding the death of Dr David Kelly (July 18, 2013).

In Stage 2 we learned lessons from Stage 1, approaching access more indirectly. We addressed two aspects of our recruitment attempts which appeared to compound the access problem. One concerned participants’ perceptions of how they and their beliefs would be characterized by the research. Another concerned the participants’ overall perception of the research and the researchers – their broader sense of our trustworthiness. These are often cited as key issues in accessing hard-to-reach samples in ethnography or other fieldwork (e.g., Norman, 2009; O’Reilly, 2009; Bengry-Howell and Griffin, 2012; Browne and McBride, 2015). While we did not engage in ethnography, our approach used methods frequent in such research.

Regarding the first, our experience in Stage 1 confirmed Wood and Douglas’s (2015) finding that CT believers resist the label “conspiracy theory,” which they take to stigmatize them and their attempts to understand the world – excluding them from “the imagined community of reasonable interlocutors” (Husting and Orr, 2007, p. 127). They instead preferred self-descriptions as being involved in “research” about how to explain unsettling events, seeking the truth about them, and thereby having an interest in “alternative explanations” or “alternative worldviews.” The use of non-stigmatizing labeling during recruitment was thus essential to communicate our aim of understanding CT beliefs from the perspectives of the participants, rather than imposing a particular perspective on them or seeking to debunk them. We thus were careful to avoid the term “conspiracy theory” during recruitment and the interviews (except when participants were themselves invited to qualify or debate its meaning and application). Additionally, to enhance trust we used descriptions CT believers employed to describe themselves. Hence, our invitation described people who were “truth seekers” or “change seekers,” who “have alternative worldviews and beliefs, and may be critical of mainstream media, politics, economics, religion, or society.”

Regarding the second aspect, our Stage 1 experience suggested that direct contact with potential participants would be difficult, since LSE is often seen as ‘part of the problem.’ We thus adopted an indirect approach, via a trusted intermediary or gatekeeper, whom the participants themselves would accept as indicating our credibility. This was achieved via the webmasters of several on-line communities in the South East of England. MH asked the webmasters to place a request for participants on their community websites. Interested members then contacted MH directly to ask any questions before committing themselves to involvement and to arrange timing and locations of interviews. This ensured that the research project was first framed within a non-judgmental context which supported the free expression of participants’ beliefs. Although indirect, the approach did not conceal MH’s academic affiliation; to withhold this information until later would likely have suggested deception and undermined the development of trust.

### Participants

We interviewed participants between July 2013 and May 2015. In Stage 1, we recruited three participants who were interviewed on July 19, 2013, in London (a 43-year-old man, hereafter R1.1), and August 4–5, 2013, and in a location outside the United Kingdom hereafter R1.2 (man, 57 years) and R1.3 (woman, in her late 40s). In Stage 2, 36 interviews took place with each of 18 participants being interviewed twice. We only report findings here from the first interview with each participant (hereafter, R1–R18), as the second interview focused on political participation in general and less on CTs. Initial interviews took place between 9 and 23 March, 2015, in Kent (*n* = 2), Central London (*n* = 9), Greater London (*n* = 1), Suffolk (*n* = 2), and via remote communication, e.g., Skype (*n* = 4). Follow-up interviews took place in the same locations in late May 2015. There were 10 men and 8 women, ages ranging from 23 to 70.

### Interviews

In both stages, we used semi-structured interviews, which outlined the research purpose, after which participants gave consent to take part; interviews were conducted in English and audiorecorded. The Stage 1 interview protocol focused on respondents’ personal backgrounds, on the ideas of David Icke, on religion and spirituality, and on contacts with like-minded others or non-believers. The Stage 2 interview protocol developed from Stage 1 and asked participants to describe how they came to be interested in alternative explanations, to indicate the kinds and range of CTs (if any) they believed, to reflect on the content of those beliefs and their connections with “new age” and other beliefs, and to indicate the kinds of social and political actions and relations they typically engaged in. Interviews typically lasted 60–90 min.

### Data Preparation and Thematic Analysis

All interviews were transcribed verbatim, but without detailed transcription of backchannel utterances, disfluencies, or other paralinguistic information. We employed thematic analysis to discover the range of contents produced by participants. Thematic analysis is widely used in the analysis of texts and transcripts, well-suited to exploring worldviews and social representations (e.g., Braun and Clarke, 2006; Joffe, 2012). It assesses and categorizes the kinds of meanings that are expressed, in a way that stays close to the texts themselves. Our thematic analysis combined both bottom-up, data-driven and theory-driven, top-down elements. Our method was abductive, the simultaneous ordering of data and emergence of a conceptual framework into a coherent logic that offers a productive guide for research.

Given the prior research on worldviews and CT rhetoric described above, we started from an initial list of theoretically relevant themes like “the nature of reality”, “the ingroup,” “the outgroup,” “the self,” or “sense of agency.” In Stage 1, we triangulated data from several sources: interview transcripts (with R1.1, R1.2, and R1.3), blogs and materials produced by CT entrepreneurs (most prominently David Icke, e.g., a DVD recording of his Wembley event, books and web discussions) and participant observations. Subsequently, the original themes were modified (and developed into more specific subthemes) in discussions amongst the authors. The outcome was an initial, informal mapping of key themes of a conspiratorial worldview. In Stage 2, the 18 interview transcripts (R1-18) were distributed amongst AB, MB, BF, and MH, who coded the interviews individually according to the themes generated in Stage 1. This procedure suggested that while Stage 1 themes also arose in Stage 2 interviews, they could not fully accommodate the range and details of contents in Stage 2. As a consequence, over a series of meetings, we refined the Stage 1 themes to accommodate new variations that emerged. This resulted in a final list of six themes:

(A) Reality: Participants’ views of reality – the causal forces in society that might sustain any CTs to which they subscribed, and whether appearances can be taken at face value or not.
(B) Self: Participants’ views of themselves – their biography and any significant events by which they became interested in CTs, and their subsequent personal development.
(C) The outgroup(s): Participants’ views of any outgroups defined relative to CTs – e.g., conspiratorial group(s), other non-conspiring members of society.
(D) The ingroup: Participants’ views of any community or ingroup to which they belonged – e.g., other CT believers, prominent individuals who act as leaders or ‘heroes’ in those communities or in the promulgation of those beliefs.
(E) Action: Participants’ CT-related actions – e.g., political engagement, social meetings.
(F) The Future: Participants’ views of how the world will be in future – based on continuation of conspiracies or on successful challenges to those conspiracies.

In analyzing the Stage 2 interviews, the variations in the way participants talked about these themes suggested potentially different depths of engagement with the contents of the CT worldview: starting from an inkling that “things are not what they seem to be” and moving toward full endorsement of a conspiracy worldview via various stages. This observation was the basis for our typology.

## Results: An Ordered Typology of Conspiratorial Mentalities

We present results as follows. In section “The Conspiracy Worldview Reconstructed”, we describe our reconstruction of the conspiratorial worldview in its fully fledged form, as an overview of our findings. In section “Typology of Individual Variations on the Conspiracy Worldview”, we describe our typology of individual variations on the conspiratorial worldview, according to the themes identified. In section “Thematic Variations”, we describe variations on each of the themes. Wherever appropriate, we reference individual interviews or include verbatim quotations from the interviews as illustrations.

### The Conspiracy Worldview Reconstructed

**FIGURE 1:**
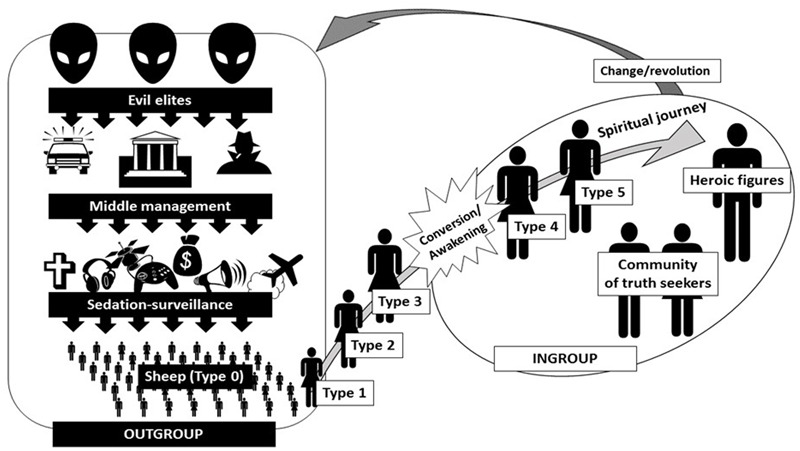
The conspiracist worldview: an elaborate hierarchy of deception and progressive degrees of insight.

A graphical summary of the reconstructed conspiracy worldview is given in **Figure 1**: this features the five main themes – the outgroup, the self, the ingroup, action, and the future, as below.

The outgroup is structured around the official narratives of events, which are illusions that hide the reality that is depicted in CTs. There are three hierarchically ordered subgroups in the outgroup. The first group is the “sheep,” the masses of anonymous people who believe in official narratives. They are dormant, being sedated by fast food, popular culture and entertainment, religion, chemtrails, vaccines, and the pursuit of normative goals like money, family and the like. They are also monitored via invasive surveillance techniques. The second group is the “middle management,” individuals and groups who occupy visible positions of expertise and power in society, including politicians, police, the military, business consultants, or scientists. They are responsible for maintaining the sheep in thrall, and they answer to the third group, the “evil elites” (Campion-Vincent, 2005). These latter are the actors who have true power: secret cabals acting in the shadows, controlling middle management to achieve and maintain world domination to further their own ends. Evil elites can be government organizations like the CIA or MI6, multinational corporations or conglomerates (e.g., Big Pharma), networks (e.g., the Bilderberg group), royalty, particular ethnic groups (the Jews) or even reptilian aliens.

The self is seen as on an epistemic and/or spiritual journey of discovery that can involve several stages. We distinguish five types of CT belief which correspond to those stages, with qualitatively different ways of being conspiratorially minded based on different degrees of elaboration of CT ideation; the process along them may indicate a path of conversion. Type 0 comprises individuals who subscribe to the official version of events, the sheep in the outgroup. The CT journey begins with unease with the way the world is, or a sense of being different or not fitting in. Interactions or altercations with sheep lead to the self being ridiculed or criticized, pushing him/her further out of the system and toward initial belief in specific CTs (this corresponds to Types 1–3). At some point, a conversion experience or spiritual awakening occurs, sometimes triggered by a traumatic personal experience (illness, loss of a loved one) or a public event like 9/11. It is at this point that the self subscribes to a fully fledged conspiratorial worldview, either postulating a conventional ontology of evil elites (Type 4) like “Big Pharma” or MI6 or a supernatural ontology (Type 5) like reptilian shapeshifting aliens. Types 4 and 5 are thus not differentiated by the extent of their conspiratorial belief but by the content of that belief.

The ingroup comprises individuals who have awakened to the reality behind CTs: like-minded truth seekers on similar research or spiritual journeys, sometimes acquiring an almost mystical sense of collective agency. Related to this community are leader figures or “CT heroes,” varying types of individual to whom participants may have different forms of allegiance – e.g., maverick intellectuals or scientists with contra-establishment views (e.g., Chomsky), gurus like David Icke, or historical figures like Christ or Buddha. This ingroup has porous boundaries interviews typically lasted 60–90 min, with other communities (e.g., hackers or the Occupy movement). Ingroup members communicate with each other *in vivo* and on-line and sometimes engage in coordinated political action (e.g., organizing protests, joining a commune).

The fully elaborated conspiratorial worldview involves a vision of the future where change will come, overthrowing the evil elites. This may arise “naturally” from increasing public awareness of cover-ups, or from additional direct actions. So the sensemaking function of conspiratorial mentality is connected to mobilizing or demobilizing political action. The personal future of the self is entwined with this macrodestiny in that it is the culmination and vindication of the journey.

### Typology of Individual Variations on the Conspiracy Worldview

The data revealed substantial variation between participants’ beliefs, which forms a typology (see **Table 1**).

**Table 1:** Typology of Conspiracy theorie (CT) believers leading to conspiracist worldview as a function of key themes.

Theme		Type 1	Type 2	Type 3	Type 4	Type 5
Reality		Something is not in order	There is more to reality than meets the eye	Some official narratives are not real	All official narratives are illusions	All of reality is an illusion; to understand real reality requires an unusual ontology

Self	Self-view	Outsider keeping an open mind	Outsider keeping an open mind	Outsider committed to a specific CT	Outsider relative to wider society, member of enlightened community	Outsider relative to wider society, member of enlightened community

	Self-development	Questioning process	Questioning process	Questioning process	Truth seeking Conversion	Truth seeking Conversion

Ingroup	Leaders	Identification/admiration General epistemic followership – role model researchers	Acknowledgment Interest in privileged source concerning specific topic(s)	Acknowledgment Interest in privileged source concerning specific topic(s)	Identification/admiration General epistemic followership – role model researchers	Identification/admiration General epistemic followership – role model researchers

	Community			Sense of community based on questioning	Sense of community based on shared CT perspective	Mystical sense of connectedness; a sense of having been initiated and awaken by an existential experience

Outgroup	Conspirators			Isolated outgroups	Outgroups linked in network, ordinary ontology	Outgroups linked in network, supernatural ontology

	Sheep			Do not see through specific cover-ups	“Asleep”, unware of being controlled by external forces	“Asleep,” unware of being controlled by external forces

Action				CT-based political action	CT-based political action; engaging with CT community	CT-based political action; engaging with CT community

The Future					Optimism conditional on revealing conspiracies: universal awakening	Optimism conditional on understanding one’s relation to the supernatural: awakening for the selected few

#### Type 1: Something Is Not in Order

One participant (R6) expressed this dissatisfaction with the status quo and mainstream problem solutions, a sense that the world is out of joint, and a desire to proffer solutions within commonsense ontology and conventional values. R6 explicitly disavows the relevance of CTs, not considering their potential truth or falsity: “I don’t mean to make that sound like there’s a conspiracy such as the Illuminati conspiracy. I am not, I don’t delve into that. Just, is there an over-influence? I don’t mean, I don’t believe that our politicians are evil people.” R6 saw themself as an “issue entrepreneur,” offering a website and criteria for developing societal solutions like “regulated capitalism” with a “greater happiness index.” So Type 1’s unease is an entirely conventional questioning of political orthodoxies, which does not see the relevance or potential truth of CTs. No particularly high degree of epistemic uncertainty attaches to this position.

#### Type 2: There Is More to Reality Than Meets the Eye

Two participants (R4 and R15) expressed this dissatisfaction with the status quo and a sense that there is really more at play in the world than appears to be the case to ordinary observers. This is broadly skeptical, aiming not to make “false negative” assumptions about reality, suspending (dis)belief pending further evidence. By contrast with Type 1, Type 2 sees CTs as relevant and possibly believable: R15 says (re 9/11): “In my opinion, the mainstream story is a load of crap but at the same time, I can’t say with any certainty what really happened. I just don’t think it is as it appears … I can’t say what has happened but I don’t believe with certainty.” And R4 suggests that the decision about whether to follow CTs is an active one: “We have the choice of what we will buy into,” and chooses not to do so because “belief in this is pretty damned sinister,” and leads “people [to] give their energy to negativity.” This uses commonsense ontology and expresses uncertainty about official explanations. R15 says, “I really hate it when people shoot down … ‘CTs’ and I’m like, ‘why, why, why did you shoot this down? Because BBC News told you that Al Qaeda flew a plane into a building.’ That to me is the definition of narrow-mindedness. I mean I don’t know what happened, I have no idea what happened … I can’t say with any certainty this did happen and this didn’t happen.” R15 explicitly juxtaposes a potentially believable specific CT with unbelievable general CTs: “Like 9/11, I think it is perfectly reasonable and not crazy to say that one is suspicious of the mainstream story and that’s fine most people can get on board with that. The minute you put David Icke into the mix with his Reptilian nonsense, you are then devaluing a whole field.” R4 also expressed open-mindedness about CT and non-CT explanations: “that middle zone of ‘I believe it and I don’t believe it.’ I don’t have to come down to one side or the other.” Type 2’s unease thus runs deeper than Type 1, accepting the relevance and the possible truth of specific CTs.

#### Type 3: Some Official Narratives Are Not True

One participant (R10) expressed this view, advancing a CT to address one specific issue, but disavowing generalized CTs. This CT used commonsense ontology and assumptions to explain the behavior of specific conspiratorial agents. R10 suggested that “chemtrails” produced by aeroplanes have not been satisfactorily explained; following investigation, R10 suggests it may connect to weather manipulation, but believes there is a cover-up. For R10 this CT belief has no monological extrapolation: for example, of the Illuminati, and New World Order, they say: “I don’t know. I’m not too familiar with that. I don’t really know what I believe about that.” However, the general uncertainty is not ameliorated – other conspiracies could be possible, though there is no clear evidence either way. For R10 this is because of a lack of accurate information: “If you are going to have a society where a lot of truth isn’t told and if there are outlets for truth-tellers why would you allow that? It would be so easy to create a misinformation site to discredit that,” created by “people who are currently in control of society.” This lack of trust in authority and its explanations does not generate monologicality: CTs apply to specific cases but are not the default frame of reference In Type 3, but even more in Type 4, participants indicate increasing concern with the deceptive nature of official narratives (Sutton and Douglas, 2014).

#### Type 4: All Official Narratives are Illusions: The Mainstream versus Reality

Several participants (R2, R8, R11, R12, R13, R14, and R16) expressed this monological conspiratorial worldview as a default frame of reference. This uses commonsense ontology of conspiring agents with, as the quasi-religious account of CTs (Franks et al., 2013) suggests, a minimally counter-intuitive understanding of their actions and agency; ordinary people and groups able to control things which are usually seen as outside human control, e.g., financial markets, climate change and variation. Supernormal agency in specific areas is ascribed to normal actors. Analogous religious representations (e.g., Sperber, 1996; Boyer, 2001) involve uncertainty because their implications are not fully processed – for example, in the Roman Catholic Mass the wine is simultaneously wine and the blood of Christ (Franks, 2003). The uncertain but potentially malign qualities of authority agents supports a mistrust of authority. For example, R2 aims to develop a “unifying theory of political economy,” to explain financial crises and governments’ complicitness in them, and explain 9/11, where “what can’t be true is an official story”; this lack of trust extends to official ‘false flags’ regarding other CTs by R2 (e.g., the murder of JFK on November 22, 1963, or the Charlie Hebdo attacks of January 7, 2015). R11 mirrors this pattern: one CT – the legal issues surrounding the United Kingdom’s decision to go to war in Iraq in 2002 – is used as the paradigm case for generalizing to others (e.g., 9/11/2001, 7/7/2005), so that ultimately, “we can no longer trust our government.” Hence, a monological lack of trust in official sources generates widespread CTs.

#### Type 5: All of Reality Is an Illusion: The Ontological-Symbolic Turn

Several participants (R1.1, R1.2, R1.3, R1, R3, R5, R7, R9, R17, and R18) expressed this fully fledged conspiratorial worldview. However, unlike Type 4, at least some of the key agents hypothesized go beyond commonsense ontologies to supernatural explanations incorporating non-human agents or human agents with non-human lineages. R1, following David Icke, speaks of alien reptilian entities which “feed on fear and lower energies which is why there is again a certain control because they are manipulating the planet.” R5 refers to contacts with UFOs, and a controlling human “cabal” originating in non-human aliens. R7 also refers to controlling aliens, but does not suggest these are reptilian nor any human contact with them. Such entities are able to demonstrate control via capacities that go beyond the human – an ontology of supernatural entities possessing supernatural agency. Whereas for Type 4 there appears an essential connection between the espoused CTs and their monological generalization, for Type 5 there is no such connection; instead, what guarantees monologicality is the appeal to an ontology populated with supernatural agency which permeates all important areas of life. Here we hear of the lizards and shapeshifters who control things behind the scenes. The distrust of authority may be a consequence rather than a cause of monologicality. The all-embracing explanation renders the CTs immune from doubt. Nor do they answer to publicly available empirical data in the way that Type 4 at least has the scope to do. As R1 comments, “It doesn’t matter if you think, ‘oh this guy that everybody is talking about is absolutely nuts,’ because it is part of my journey of understanding my existence.”

#### Summary of Typology

Several key points arise from this typology. First, not all CT belief is monological – it is possible to entertain or believe in single CTs (Types 2 and 3) but reject others and not subscribe to a full-blown conspiratorial worldview (Types 4 and 5). Second, some monologicality may derive from a lack of trust in official explanations (Type 4), but other cases derive from an all-encompassing supernatural explanation of reality (Type 5). Third, overlaying this typology appears to be a curvilinear pattern of degree of epistemic certainty. The end-points of the typology express epistemic “closure” or certainty. Types 0 and 1 involve an acceptance of the commonsense ontology of agents and causes that frame official narratives. Type 2 uses the same ontology but adds doubt in questioning whether all official narratives are true. Type 3 offers more doubt in explicitly distrusting specific official narratives, but expresses closure in adhering to single CTs. Type 4 involves generalized distrust of official narratives, introducing doubt as part of an overarching conspiracy worldview. Type 5 involves a supernatural ontology that reframes official narratives in its own terms – here, distrust of authority is framed by a lack of doubt about how to explain its untrustworthiness (i.e., the supernatural ontology). Such a general curvilinear pattern of epistemic certainty suggests that CTs are likely to succeed most clearly in symbolic coping or anxiety reduction when they are part of a monological worldview – single CTs seem likely rather to exacerbate anxiety. Fourth, Types 4 and 5 participants expressed complex sets of interconnected beliefs in which there was no blanket rejection of authority or embracing of all CT-supporting evidence – they were suspicious not only of authority but also (though to a lesser degree) alternative explanations; moreover, beyond merely denying authorized explanations, they advanced complex narratives about the interconnections between specific conspiracies (e.g., using the differentiation of the outgroup in **Figure 1**). The worldview that underpins monologicality, for our participants, goes beyond the denialism or “closed” mind (Goertzel, 1994) often expressed in the literature. Our observations thus suggest that monologicality is not a defining feature of belief in conspiracy, but a variable end-point on an escalating spectrum of conspiracy-mindedness.

### Thematic Variations

#### Reality and Ontology

Sutton and Douglas (2014) suggested that a potential underlying feature of monologicality is the deceptive nature of official narratives. This corresponds in our interviews to different positions on the nature of reality. Broadly, the nature of reality is unproblematic for Types 0–3 participants, even though uncertainty is expressed as to some anomalies. Some Types 4 and 5 participants expressed variations on the theme that the fabric of everyday reality is an illusion which is intelligible only to the selected few, similar to the Platonic cave allegory (R16) or films like “The Matrix” (R16). Some (Type 5) opined that “real” reality is beyond the three-dimensional world of the five senses, to be sensed by engaging in practices like meditation or reiki (R10 and R16). Such engagement involves mystical experiences like feeling energy flows or developing a collective consciousness: “in what the Vedics call the Cochic record, the cloud hard drive in the sky that we all share, so each of us has a folder in that hard drive that we record our memories, thoughts and feelings and emotions and whatnot. And as long as you’re in this life form and you’re held with the five senses in the three dimensions in the time-based worldview, then all you can ever recall is anything you’ve written in your own folder. But at some point you become capable of receiving information from the whole cloud” (R17).

#### Self-view and Self-development

Many participants explicitly rejected the label “conspiracy theory,” corroborating findings of Wood and Douglas (2015). This rejection was independent of participants’ positioning in the typology. Some suggested the label is used to deliberately undermine alternative explanations, since it categorizes together both “reasonable” alternative explanations using everyday ontologies for single events and more complex conspiratorial narratives using more novel ontologies (e.g., R1.1, R5, and R11). The evident oddity of novel ontologies is used by association to undermine reasonable explanations (see Husting and Orr, 2007). The CT label may also diminish the force of the argument by shifting the focus onto the credibility of the CT believer (R10, R11, and R7). R15 suggests, “I think unfortunately, these things do get lumped together, these alternative viewpoints or CTs, everything gets lumped together. And when you have got some crazy man like David Icke spouting nonsense all the people who are then saying quite sensible things, all get lumped together as loony, tin-foil crazies.” One suggested the label ‘conspiracy theory’ was coined by the CIA in the 1950s to discredit people inquiring into governmental “black ops” activities (R2). Another suggested the label was a way to shut down a potentially illuminating conversation: “It’s become … a weapon to close down the conversation. “Oh, so you believe in CTs, do you?” Where can you go with that? Quite often it’s a putdown” (R1.1). As a consequence, participants preferred to describe themselves as researchers who are “seeking after the truth” or knowledge, or developing alternative explanations (R8, R1, R15, R9, R13, and R11). As R11 says, “I am always after the truth. It’s probably why I am considered a conspiracy theorist.”

When asked how their interest in alternative explanations began, many cited a gradual progression based on a combination of personal issues and “projects,” which had been expedited by their experiences of major public events. Types 4 and 5 participants referred to early experiences of being different (R1.3), traumatic personal experiences such as severe illness of self or a close other, or bankruptcy and homelessness (R3, R12, R13, R9, R18, and R11); some reported specific exceptional episodes such as out of body experiences, experience of UFOs and aliens (R9 and R16). Against this backdrop of general questioning of reality, specific public events were often referred to by Types 4 and 5 participants as catalysts for their interest in CTs – e.g., 9/11/2001 (R2: “9/11 did it for me”; R17, R12, R7, and R11), the financial crash of 2008 or the Iraq War (R2 and R7).

Some participants (independently of type) referred to family problems and enmeshing social relationships (R7 and R4), and others to an interest in meditation and psychological processes (R5, R10, R1, and R3). These appear to have progressively triggered questioning of received explanations. For some, this generated an intensive interest in philosophical questions or ‘mysteries’ (R7, R16, R4, and R10) – e.g., R16 wanted “to be part of the 1% that understands Plato’s cave.”

These findings are striking: to understand why CT thinking is “sticky” (i.e., an attractive and persuasive way of thinking, which resists change), we may need to consider not only its content and role in individual and social anxiety reduction. We also need to consider how CTs emerge from the personal-development, epistemic and social-political “projects” that first led people to consider alternative explanations. Some such projects suggest a gradual process punctuated by “conversion” episodes leading to Types 4 or 5 monological outcomes, consonant with the quasi-religious approach (Franks et al., 2013).

#### The Outgroup: The Conspirators and the Majority

Participants described a structured outgroup. Types 2 and 3 participants typically referred to only two subgroups: the conspirators and the controlled majority. They also expressed uncertainty about whether the conspirators really performed all of the alleged actions. Thus, R15 (Type 2) suggested a role for United States governmental and military organizations in covering up their own deliberate engineering of major events (such as Pearl Harbor, 9/11) to deflect attention away from their real activities and to legitimize attempts to further extend their reach. R10 (Type 3) mentioned that some people have beliefs about the Illuminati aiming to establish a New World Order, but “I’m not really sure.”

Greatest differentiation of the outgroup was offered by Types 4 and 5 participants, who differentiated three subgroups: the wider controlled, non-agentic class who believe in conventional explanations (“sheep” in **Figure 1**), plus two parts of the “ruling class.” The “evil elites” (**Figure 1**) are hidden agents who define the ends and nature of the conspiracies, and the mid-level proximal agents (“middle management” in **Figure 1**) are observable actors that provide the means for the elite groups’ ends and promulgate the authorized explanations.

This results, for some (R1, R3, and R12), in a society managed by fear. Middle management (e.g., governments, armed forces, police and the “Big Four” accounting firms) engage in conspiracies that are understood by ordinary conventional explanations, using everyday ontology and possessing commonsense qualities (Type 4). But these are merely the agents of the real elite. R3 suggests that governments are “puppets,” the police are “minions” and the real rulers are in the shadows. Others gave examples of conspiracies – e.g., in dealing with pedophilia in establishment circles, the United Kingdom Prime Minister was sidestepped (R14), and one or two figureheads from middle management were “sacrificed,” allowing allow the evil elite to maintain its conspiracies whilst giving the appearance to the sheep of rooting out the culprits (R1). Middle management is thus a buffer against real change, even when apparently held to account.

The true evil elites are hidden from view, possessing qualities that depart from ordinary ontology to varying degrees, often viewed as shadowy entities with mythical histories. Most are understood in terms of powerful families with associated bloodlines or religious dynasties with associated forms of initiation and membership, such as the United Kingdom Royal Family, the Rothschilds, Rockefellers, Bushes, Clintons, all of whom are interconnected with the Holy Roman Empire, Saudi Arabia (R16, R2, R9, R13, and R12), or the Illuminati (R1 and R5). Together they form a “structural power elite, with interlocking structures” (R2), closed to all outsiders. They share an aim for self-replication in the pursuit of power and the maintenance of control – at any cost to human life.

As R17 comments, “fish rots from the head downward”: evil elites control the establishment, setting up educational, industrial, financial, and governmental institutions to serve their malign aims in controlling what the sheep do and believe. In Type 5, the account of evil elites appeals to non-commonsense ontological assumptions about agents – e.g., the elite really are “reptilians” (R1), directly expressing Icke’s “alien lizard” iconography (Lewis and Kahn, 2005); or they have a lineage in other alien life forms and UFOs (R5, R7, and R17). For some, this issues in the sweeping metaphor that “earth is a slave ship” (R5) or a “prison planet” (R1) controlled by those aliens or their descendants.

When talking about the sheep, Types 4 and 5 participants in particular referred to middle management’s “sedation” of the populace – e.g., via alcohol, entertainment (football, TV, the royal family, fast food), and having to work hard and be self-interested in order to make a living (R1.1, R12, R9, R3, and R7). As R7 put it, “Yeah, you know the ‘normies’ is what I call them, people going to work, doing their job, not questioning anything, but all they’re doing is spending money on the system and keep it circulating so that banks are corporating the money.” The sheep are thereby happy to be part of a passive, ignorant “herd” or “hive” (R9, R13, R14, and R12), leading to a state of “collective unconsciousness” (R9). Interactions with sheep about CT-related topics often led to conflict or ostracism (R1: “try to ridicule you and try to convince you that you have lost the plot”), requiring them to be circumspect in raising such topics (R1.1: “Most of the time, people don’t want to listen, you have to get people into a certain mental space for them to listen properly”) or even to progressively avoid them.

All participants referred to the mass media as a significant part of the control process: filtering information, leaving out important issues and presenting infantile, sound-bite journalism and entertainment (R1, R3, R4, R5, and R10). The media sets the agenda for what can and cannot be discussed; as R15 put it, the “dog doesn’t bark, the journalistic machine doesn’t work, politics is broken”, perhaps because as R5 notes, the mass media worldwide are “controlled by the same three or four organizations.” However, alternative media may be no more reliable: “I would say for every conspiracy theory ‘theory,’ there’s an equal number of misinformation sites” (R10).

#### The ingroup: Truth Seekers, Awake and Connected

Regarding ingroups, participants reported a mixed picture, independent of CT type. Developing CT interests, for some, created difficulties in their relational ingroups: family, friends, or colleagues mocked or discredited their views (R1, R7, R14, R18, R3, and R15), leading them to be reluctant to discuss the issues with them. This led some to seek social contacts with other people interested in CTs. Their on-line and *in vivo* contacts generally involved developing alternative explanations (R3: “helping you connect the dots by talking together”), by talking with like-minded people who are critical, open-minded, anti-establishment and well-read (R2, R10, R14, and R15). The social connections are key to reinforcing, rehearsing and maintaining CTs, with some noting that group membership had made them “more convinced” of CTs (R7), perhaps because it “keeps you knowing” (R14).

What connects the CT ingroup is that they are all truth-seekers, and “It’s wanting a fair and just society, and what wakes people up? I don’t know, you are either asleep or awake because once you are awake, you can’t ignore it” (R12). The metaphor of truth-seekers being “awake” (a recurrent motif in Icke’s writings) compared to the “sleeping” sheep, was widespread (R1, R2, R4, R9, R10, R12, and R14). Other expressions also differentiated the ingroup from the controlled class – “we” have greater “awareness” (R3, R5, R8, R13, R14, and R17), or “our” eyes are “open” and theirs are closed (R3, R7, R9, R15, and R18). As with all in-group communication, the informational and the affiliational overlap (Enfield, 2006). There were two significant aspects to this. First, participants refer to the groups as offering a “community” (R17, R3, R4, and R8), a “spiritual side” (R8) which supports collaboration and connections that “express humanity,” helping people to “wake up, get back to connecting rather than atomized” (R4). Second, for some, the group offers a sense of positive, collective agency, which could substitute for the failed or inappropriate agency of governments and media. In the groups, they “realize that we are more than we think we are collectively” (R17), and “collectively there’s a quickening, raising of awareness, things ain’t right” (R17), so that the group identity has the common denominator of a positive outlook in the battle for social change (R4). In the groups, R14 suggests, “you feel like you could make a change because everyone felt like that, we could make a change because we have the power because we are the consumers. We could break down the 1% if we all agreed”. More succinctly, R5 expressed this as “we are a supreme minority, but growing.”

Although our Types 4 and 5 participants all saw themselves as connected to communities or groups affiliated around a general CT stance, Type 5 added a pan-human dimension based on their spiritual beliefs: “We are an organism collectively” (R17), or ‘I believe that somehow we are all connected universally.” Some saw this as the origin of their beliefs (e.g., R5: “I get these knowings. I know things is all I can say and I guess it comes from some sort of universal collective wisdom”), or as a basis for understanding themselves (e.g., R1: “It’s like everybody is on the same journey, same path, but people are at different stages”).

But group membership also brings the possibility of schism, and two participants expressed unhappiness at the restrictions of previous groups. One indicated disenchantment with the Occupy Movement, which led them to be less active in meet up groups, and to limit interaction to only “talking” to national and international groups, principally on the internet: (R2). Another (R9) was frustrated that the meet up group’s committee structure “paralyzed” discussions, effectively censoring the range of CTs and issues canvassed.

One key aspect of reinforcing beliefs and norms, and supporting ingroup coherence, is the relation between a group and a leader (Haslam et al., 2011): more successful (and more positively evaluated) leaders are often seen as highly prototypical or ideal members of the ingroup. Our participants had a highly developed sense of who were the prototypical – leading – truth seekers, and often deferred to their knowledge and referred to them in discussion. Some were domain-specific – “maverick” scientists with expertise in areas like chemistry, physics and archeology (R2, R9, R10, and R18), or economics and politics (R1, R2, R13, R6, and R14); such references often used rhetorical devices indicating the arcane knowledge at stake and positioning the CT believer relative to the interviewer: for example, “have you heard of …?” (R2). Others – particularly Types 4 and 5 – were more general, identifying with or admiring a key figure as a prototypical truth seeker or CT researcher; many cited David Icke as an inspiration in uncovering conspiracies long before other people (R1.1, R1.2, R1.3, R1, R2, R3, R5, R8, R16, and R17). For example, R3 suggested that David Icke “caused me to expand my way of thinking and join dots and put this and that together.” However, some acknowledged mixed feelings about the respectability of some of his material (R9 and R17), even though “not one of his books has ever been challenged or faced with a law suit” (R5). Others also cited figures from popular culture (e.g., Russell Brand: R10, R12, and R14) or those who run CT-focused internet sites (e.g., Alex Jones: R1, R6, and R9). Some also referred to historically “great figures” who revealed hidden wisdom – marking the lineage of the ingroup as part of a long history of being critical of and vilified by the mainstream: the Buddha (R1 and R3), Jesus (R3), Gandhi (R1 and R14). Others cited major intellectual figures, again indicating the apparent reasonableness of their own stance: Orwell (R1 and R17), Marx or Chomsky (R13). Participants typically cited individuals who have accepted the threat to worldly status associated with challenging the status quo, making what Henrich (2009) calls CREDs (credibility enhancing displays) in which CT declarations gain extra force by their declarers’ paying the costs of exposure to public opprobrium. Public vilification amounts to proof of concept. Such people are therefore respected for epistemic reasons but also admirable for personal and moral reasons. This is another aspect of the conspiracist worldview consonant with quasi-religiosity. Interestingly, whilst most of our participants expressed admiration for fearless researchers and respect for their empirical work, the strategy of placing *oneself* in such an illustrious lineage (epistemic and personal identification) was used only by participants of Types 4 and 5 and Type 1 (R6) – evidently, for quite different rhetorical and epistemic reasons.

We note three implications of deference to CT leaders: first, it is selective – CT believers employ many information sources, and few accept everything the leaders promulgate (they retain a degree of criticality even about their heroes); second, they are conscious of those leaders’ public credibility and of the need to persuade others of the reasonableness of their own stance. Third, such deference varies according to CT type.

The connection with other believers in CTs (whether in promoting ingroup identity or developing ingroup schisms) challenges the notion that CT belief is inherently socially disengaged: rather, there is a sense of wanting to re-make society and the inchoate hope that being involved with other CT believers may contribute to this.

#### Political Action

Our follow-up interviews in Stage 2 took place just after the time of a United Kingdom General Election in 2015. When asked, 13 participants reported having voted in the election (and voting/not voting was not connected to particular CT type). Participation in other forms of political activity was varied and unconnected to the typology. Some talked of general, sweeping political aims. For example, R7 asserted, “Political systems in every country need to be abolished and redone,” whilst R4 mentioned the aim to “transform representative politics into enactment politics” – direct action replacing voting. Others engaged in more specific activities – for example, attending meet ups (R3 and R17), setting up websites (R6) or writing books on relevant topics (R3 and R 6). Whereas some were involved in organizing demonstrations and meetups (R12 and R17), others expressed a sense of powerlessness concerning political action (R11, R16, and R15) – for example, taking part in demonstrations is the least effective form of political action, and so is encouraged by governments: “if you can get people marching, demonstrating, protesting, it keeps them focussed on ineffective action. And the real effective action is financial action, legal action or political action, but none of that happens if they go on a demonstration” (R11). Others suggest that the potential for successful political action is vitiated by the very nature of the ingroup (R2, R9). For example, R2 stated, “my main frustrations with Occupy and all these other activist organizations that we could quite easily win if we addressed and stuck to the main causes. In other words we presented a coherent narrative but you have all these demonstrations talking about symptoms and they never come together and so they are ineffectual in that sense.” Taken together, this theme suggests that embracing CTs does not necessarily entail political cynicism or disengagement from all democratic processes; rather, CT believers appear potentially engaged in politics and citizenship but skeptical about the available means in conventional politics. Thus, the conspiratorial worldview might relate to *prefigurative* political or social mobilization, in particular the imaginary construction of “alternatives,” with little account of detailed means for achieving that goal (see Yates, 2015).

#### The Future

The question of the future concerned the degree of optimism about possible personal and collective change. The broad pattern was intuitively paradoxical: the more monological our participants were, the more optimistic they seemed to be, though that optimism was contingent. For Type 4, a non-conspiratorial future was contingent on the discovery and public knowledge of the conspiracies in the political world: when everyone wakes up, the political world would be transformed into a non-conspiratorial world [paralleling Byford’s (2011) “naïve optimism”]. For Type 5, the positive future was more contingent on individuals coming to understand their relation to the supernatural forces that govern the universe: self-discovery allows coming to terms with those forces, though not thereby removing the supernaturally based conspiracies. Monologicality thus leads to the possibility of major future change. By contrast, those with a less monological worldview tended to be less optimistic about the future, because each conspiracy must be assessed and challenged on its own terms. Type 3 participants, for example, see the likelihood of change as restricted by the reality of specific, concrete conspiracies and their entanglement with power relations.

## Discussion

### Monologicality and the Spectrum of Conspiratorial Worldviews

Monologicality designates the empirical tendency for belief in one CT to be correlated with belief in others. Explanations of monologicality often invoke a nomothetical or “closed” mindset (Goertzel, 1994) whereby mutually supporting beliefs about the nature of the world are used to interpret public events as conspiracies. But research on monologicality typically has little discussion of the content of beliefs and reasoning from the standpoint of the CT believers. This in part arises from the access problem (Wood and Douglas, 2015): CT believers are averse to being researched because they often distrust researchers as “part of the problem.”

Our study investigated the symbolic resources underlying monologicality by reconstructing a conspiracy worldview – an escalating set of beliefs held by CT believers about important dimensions of ontology, epistemology, and human agency (Koltko-Rivera, 2004). To do this, we analyzed media documents, conducted field observation, and engaged in semi-structured interviews, using a variety of strategies to overcome the access problem. We described six main dimensions of such a putative worldview: The nature of reality and its ontology, the description of self, the outgroup, the ingroup, action, and the future. Patterns of positions on these dimensions led us to construct a typology of five types of escalating CT believers. Our findings converge with prior explorations of the content of CT beliefs: Some are similar to Byford’s (2011) generic “anatomy” of CTs, derived from the analysis of documents, cultural artifact and mass media sources. However, we also discovered novel aspects of the conspiracy worldview. Perhaps most surprising concerned the ingroup, which was structured and subtle, embracing both epistemic and affiliative dimensions. Byford does suggest that from the 1960s onward, “conspiracy theory became a call to mobilization, inspiring readers to gather ‘evidence,’ share it with others and become part of a community” (p.67), but there is little detailed analysis of such community. This is perhaps unsurprising, since his data sources (written outputs expressing CTs) do not offer straightforward means of assessing relations to group membership and identity. Moreover, we find evidence of a leader-follower relation, with experts viewed as “hero” researchers, fearless in their critical inquiry and uncovering of unpalatable truths. Another novel finding is the trajectory toward becoming a CT believer – a personal journey of conversion or development. For our participants, this was key to why they believed in CTs, and the type of CT to which they subscribed. Final novel aspects of our findings concerned the connection between participants’ beliefs in CTs and their tendency to engage in political action (as opposed to being disengaged from the political process), and their belief in the possibility that such action could lead to a positive future (as opposed to being cynically resigned to there being no possibility of a non-conspiratorial world).

All of these novel aspects – the sense of community, the pantheon of leaders, the personal conversion journey, the link to political action, and the optimistic future – are at odds with the typical image of monological CT believers as paranoid, cynical, anomic, irrational individuals (Douglas et al., 2016). Instead, the CT worldview may be the underpinning of a nascent social movement, prefigurative political mobilization, or at the very least an inchoate, but distributed community of engaged citizens, albeit with alternative beliefs. In this respect, our findings echo Waters’s (1997) finding that African American CT believers were better educated, more politically active, and more socially engaged that non-believers. Our findings can also explain the otherwise incongruous results (Swami et al., 2010, 2011, 2014) that CT believers may be more open to experience and more strongly support democratic principles than non-believers.

### Limitations

Our study has some limitations. First, the sample of interviews is rather limited in size, geographical location and political-ideological bent – all of which limit generalizability of our findings. We interviewed only a small number of people and mostly in the South East of the United Kingdom. Perhaps the most serious is the political bent: the majority of our participants were on the left of the political spectrum, self-identifying as interested in “alternative” explanations. It is unclear to what degree the elements of the conspiratorial worldview would hold equally for a similar sample of the milieu on the political right. Is the CT worldview of right-wing CT believers fundamentally different? There is little research on this issue, but some data suggest that it may not be. A report on the right-wing milieu in Germany commissioned by the Amadeu Antonio Foundation (Rathe et al., 2015) suggests that many elements may be similar; other analyses also suggest that right-wing conspiracy worldviews are analogous in their ingredients to what we have documented, although they may focus more systematically on Zionism in their characterization of evil elites (Byford, 2011; Imhoff, 2015).

The small sample suggests caution in a specific aspect of our interpretation: most of our participants cluster in Types 4 and 5, and fewer in Types 1–3. As noted above, our data coding combined both bottom-up, data-driven elements (hence all of the novel findings above), and top-down, theory-driven (or past findings-driven) elements. The latter grounds our postulation of Types 1–3: they are internally consistent patterns of response to destabilizing issues, which reflect different degrees of dissatisfaction with official stories. They also allow us to make coherent the idea of a conversion-related trajectory from skeptical conventional thinking (Type 1) through specific CT beliefs (Type 3) to monologicality (Types 4 and 5).

Notwithstanding this, our findings broadly converge with Byford’s (2011) analyses based on a broad set of political documents and commentary, with perhaps those concerning the sense of community afforded by CT believers and the nature and role of Type 3 requiring further empirical substantiation.

### Accessing CT Believers and other Methodological Issues

Our research approached the problem of gaining access to CT believers using methods from ethnography in two ways, both of which are time-consuming and painstaking. One was to gain trust of participants during recruitment by avoiding stigmatization through terms like “conspiracy theory” and adopting an open-minded attitude to their beliefs. The second was to increase the credibility of the research team by recruiting participants via gatekeepers who were themselves already in a position of trust.

Our results suggest that findings of quantitative, questionnaire-based studies of CTs may benefit from being supplemented by qualitative studies that seek to uncover nuances of the contents and social implications of CT belief. In this way, a rounded view of conspiratorial worldviews may be achieved. Content-wise, the kinds of CTs offered for assent or dissent in questionnaire studies typically fall under our Type 3 or Type 4, which deploy everyday ontology often with specific claims of supernormal agency on the part of the conspirators. The result is a classic, negative monologicality. There is, interestingly, little quantitative investigation of possible CTs *based on* supernatural ontologies, though CT belief itself has been found to correlate with non-conspiratorial supernatural beliefs (Darwin et al., 2011; Swami et al., 2011).

### Conspiracist Worldviews and the Quasi-Religious Approach to CTs: Research Directions

The quasi-religious approach to CTs (Franks et al., 2013) suggests that CTs can – to differing degrees – function in a manner analogous to religious beliefs, which may suggest that some CT belief should correlate with actual religious or supernatural beliefs (as for Type 5 participants). Belief in CTs might more generally be based on the form of religiosity called “quest” (Batson et al., 1993). This reflects a search after meaning that poses existential questions, regards doubting and skepticism as positive forces, and allows that answers to those questions may be tentative and partial – perhaps connecting to agnosticism (e.g., Donahue, 1985), or a cognitive style expressing symbolic doubt, rather than a specifically religious motivation (e.g., Neyrinck et al., 2010). For many of our participants, this is apt: CTs and alternative explanations are a secular “quest,” which may or may not end in belief in supernatural ontology (Type 5) or in ascribing exceptional agency to conspiring groups (Type 4).

So in our view, monologicality may be less a defining feature of believing in CTs, but rather a variable consequence of the set of CTs believed. To the extent that the set of espoused CTs grows, a degree of monologicality may emerge – but built on two foundations. One is a negative claim (rejection of official explanations, as in many past findings), and the other is a positive claim (Type 5 imputing of supernatural agents with exceptional agency, or Type 4 imputing of exceptional control or power to the conspiratorial group (Franks et al., 2013; Douglas et al., 2016). Together they express a clear conviction of our participants – that CTs may not merely express cynicism and disengagement from the status quo, but also involve positive attempts to understand and explain events leading to prescriptions for political action.

Secondly, the approach also offers a substantive view of how people can represent apparently contradictory CTs (see Wood et al., 2012). As for many religious representations, the element of uncertainty means that the content of CTs may not be fully explicated by believers: Types 4 and 5 CTs represent conspiring groups with greater-than-natural agency, but precisely what those qualities are and how those agents actually operate may remain unclear. Contradictions between CTs may not be detected or experienced as contradictions by believers, because the representations of the CTs do not have precise interpretations, and so do not support a sharp sense of conflict. They may thus permit coexistence of apparently contradictory knowledge systems (Legare and Gelman, 2008) or cognitive polyphasia (Jovchelovitch, 2006; Falade and Bauer, 2017), in which aspects of conventional and conspiratorial worldviews offer complementary, rather than competing explanations of destabilizing events.

Thirdly, our findings and the quasi-religious approach suggest further investigation of the active social cognitive processes of ‘bricolage’ of CT beliefs (e.g., how far they follow the heuristics suggested by Sunstein and Vermeule, 2009) – since CT believers are not only choosy with the contents that they draw from official sources, but also with content from alternative sources.

Fourthly, as noted our typology requires further investigation: how robust is it across participants with different demographic and political profiles? And, delving inside it, are there quasi-religious conversion processes from Type 1 where the world seems out of joint to Type 5 where reality is perceived as an illusion and an alternative ontology is invoked? What psychological processes lead from domain-specific distrust of authority (Type 3) to domain-general distrust (Type 4): when and why does the request for positive reasons to trust official explanations transition into an assumption that no reasons can be provided? It seems likely that group membership plays an important role here; this is a core feature of our Types 4 and 5, but is absent from the others. When and why does dissatisfaction with ordinary explanations (or with general CTs using ordinary ontologies – Type 4) transmute into the positive espousal of alternative ontologies in monological CTs – Type 5? This may have its root in the specific type of inquiry that characterizes the person’s quest – for example, a more political issue of control versus a more personal issue of identity. Answering such questions may require longitudinal studies focusing on the personal developmental trajectories – in biographical–idiographical terms – of CT believers.

Fifthly, the social and inter-group relations also warrant further investigation. The role of the internet in sharing and discussing CTs has been emphasized recently (e.g., Wood and Douglas, 2013, 2015). Our research demonstrates that the internet is also important in developing and supporting *in vivo* social relations and social group and social identity formation. CTs are not static, but rather dynamic beliefs that relate to individual life projects and to social behavior of various kinds. We found communicating about CTs can underpin definition and critique of outgroups, as in past research, but also formation of coherent ingroups with implications for social identity (Jovchelovitch, 2006). This suggests a further potentially fruitful area of research – how do such groupings form on-line and offline? How do individuals move from blogging on-line to meeting *in vivo*? What are the social functions of CTs, as well as their social consequences? What role do on-line and off-line group leaders perform in curating CT beliefs, and how do they relate to the ideas publicized by more widely known “CT heroes”?

Sixth, our findings suggest broadening the canvas of political actions relevant to CTs. Our participants held nuanced views of political action and – whilst some were cynical about its effect – most saw a requirement of their self-representation as “truth seekers” as putting forward concrete positive explanations and proposals for change.

Seventh – practically, our findings may have implications regarding addressing the consequences of CT belief. They suggest the need to consider not only the degree or strength of CT belief but also its content, in entertaining any practical measures to address problematic consequences of a CT worldview. Location of CT believers in our typology might, for example, moderate the impact of educational measures to combat potential effects of negative CTs (Douglas et al., 2016), or the engagement with CT social groups and leaders to ameliorate CT-related political extremism (Bartlett and Miller, 2010).

Eighth – methodologically, our typology might be the basis of an alternative quantitative measurement of CT mentality, one which involves the detailed symbolic resources of CTs rather than (as in more typical, Likert-like survey methods) sketches of those resources to measure their connection to other variables. Our typology is based on specific configurations of values of the key features of reality, self, ingroup, outgroup, action and the future. One construal of our Types 1 and 5 CT beliefs is that they combine the least elaborated and most highly elaborated conspiracist ideation, respectively, and between them there is a continuum of elaboration. Such a relation between a hierarchy of specific beliefs and an emergent continuum is typically discussed as a Guttman-type approach to developing a unidimensional scale for beliefs. This approach has been taken by researchers in the sociology of religion (Michelat, 1991), and might find some utility in quasi-religious conspiracy beliefs.

A final question that arises is: if at least some CT belief is not monological, how should it be described? What is the alternative to monologicality? Returning again to Goertzel (1994: p. 740), he claims, “Dialogical belief systems engage in a dialog with their context, while monological systems speak only to themselves, ignoring their context in all but the shallowest respects.” We used the subsequent empirical operationalisation of monologicality as the idea that belief in one CT is predictive of belief in (many) more, and found that even our most monological participants (Types 4 and 5) were still dialogical in Goertzel’s terms; that is, they utilized non-conspiratorial symbolic contents in the framing of their CTs and drew selectively and critically on both conspiratorial and non-conspiratorial evidence in their justification. Future work might investigate the possible connections with the contrast between monological and dialogical approaches to communication (Linell, 2009), which is likely to be important to the social sense-making function of CTs, as part of understanding how CT believers manage the coexistence of conspiratorial and conventional beliefs in explaining events.

## Conclusion

Conspiracy theories are widespread and important cultural forms of mind that enable symbolic sense making about threatening events or situations. Past quantitative research has suggested that CT belief may be monological, such that belief in one CT is predictive of belief in others. We sought to investigate the symbolic resources that form the contents of such beliefs by carrying out qualitative interviews with people who espouse them; in doing so, we developed an approach to address the difficulty of accessing the population of CT believers. Our results confirm, augment and to some degree challenge past findings, suggesting that different elements of CT beliefs coalesce to form a distinctive conspiratorial worldview, within which particular patterns form an escalating typology of CT mentality, only some of which are monological. Monologicality is thus not a defining feature of conspiratorial mentality, but only a special case. This finding and our concept of CT as quasi-religious lead to new directions for future research and possible methodological and practical implications.

## Ethics Statement

This study was carried out in accordance with the ethical guidelines of the British Psychological Society. The design and protocol were approved by the Ethics Committee of the Department of Psychological and Behavioral Science at the London School of Economics. All participants gave their written informed consent. All participants gave written informed consent in accordance with the Declaration of Helsinki.

## Author Contributions

BF, AB, and MB developed main conceptual framework. BF, AB, MB, and MH analyzed the data. MH collected 85% of the data, and MN collected 15% of the data. BF wrote first draft and produced final draft. AB edited first draft. MB edited final draft.

## Conflict of Interest Statement

The authors declare that the research was conducted in the absence of any commercial or financial relationships that could be construed as a potential conflict of interest.

## Footnotes

1 www.davidicke.com
